# Endophytes and Plant Extracts as Potential Antimicrobial Agents against *Candidatus* Liberibacter Asiaticus, Causal Agent of Huanglongbing

**DOI:** 10.3390/microorganisms11061529

**Published:** 2023-06-08

**Authors:** Jessica Dominguez, Krishnaswamy Jayachandran, Ed Stover, Joseph Krystel, Kateel G. Shetty

**Affiliations:** 1 Department of Earth and Environment, Florida International University, Miami, FL 33199, USA; jdomi022@fiu.edu (J.D.); jayachan@fiu.edu (K.J.); 2 United States Department of Agriculture/Agricultural Research Service, Ft. Pierce, FL 34945, USA; ewstover@ufl.edu (E.S.); joseph.krystel@usda.gov (J.K.)

**Keywords:** citrus greening, *Candidatus* Liberibacter asiaticus, antimicrobial activity, endophytes, medicinal plants, psyllid homogenate assay

## Abstract

Huanglongbing (HLB), also known as citrus greening, is an insidious disease in citrus and has become a threat to the sustainability of the citrus industry worldwide. In the U.S., *Candidatus* Liberibacter asiaticus (*C*Las) is the pathogen that is associated with HLB, an unculturable, phloem-limited bacteria, vectored by the Asian Citrus Psyllid (ACP, *Diaphorina citri*). There is no known cure nor treatment to effectively control HLB, and current control methods are primarily based on the use of insecticides and antibiotics, where effectiveness is limited and may have negative impacts on beneficial and non-target organisms. Thus, there is an urgent need for the development of effective and sustainable treatment options to reduce or eliminate *C*Las from infected trees. In the present study, we screened citrus-derived endophytes, their cell-free culture supernatants (CFCS), and crude plant extracts for antimicrobial activity against two culturable surrogates of *C*Las, *Sinorhizobium meliloti* and *Liberibacter crescens*. Candidates considered high-potential antimicrobial agents were assessed directly against *C*Las in vitro, using a propidium monoazide–based assay. As compared to the negative controls, statistically significant reductions of viable *C*Las cells were observed for each of the five bacterial CFCS. Subsequent 16S rRNA gene sequencing revealed that each of the five bacterial isolates were most closely related to *Bacillus amyloliquefacie*ns, a species dominating the market of biological control products. As such, the aboveground endosphere of asymptomatic survivor citrus trees, grown in an organic orchard, were found to host bacterial endophytes capable of effectively disrupting *C*Las cell membranes. These results concur with the theory that native members of the citrus microbiome play a role in the development of HLB. Here, we identify five strains of *Bacillus amyloliquefaciens* demonstrating notable potential to be used as sources of novel antimicrobials for the sustainable management of HLB.

## 1. Introduction

Citrus greening is the most devastating disease in citrus worldwide. It is an infectious disease that affects all species of citrus and, at present, remains incurable. To date, there is no treatment [1,2] nor resistant cultivar [3] that can effectively control the disease. In the U.S., *Candidatus* Liberibacter asiaticus (*C*Las) is the bacterial pathogen that is associated with HLB [4,5]. *Candidatus* Liberibacter asiaticus is an unculturable, Gram-negative, phloem-limited bacterial pathogen, vectored by the sap-sucking insect, *Diaphorina citri*, commonly known as the Asian Citrus Psyllid (ACP) [1]. Characteristic symptoms include blotchy mottle leaves (yellow asymmetrical patterns), increased fruit drop, and dieback [1,6]. Once infected, *C*Las compromises the plant’s vascular system by plugging the phloem tissue, which disrupts the natural flow of nutrients; this gradually weakens the host and eventually results in dead or unproductive plants [1,7]. In the United States, HLB was first detected in Florida in 2005 [1], and by 2010, every citrus-producing county in the state was infected with HLB [8]. In a matter of 14 years, total citrus production declined by 80%, and the 30 major citrus processing plants in the state dwindled to only seven [5].

With no cure or effective treatment, growers have adopted a wide range of strategies in the attempt to manage HLB. The principal method used is vector control, which is primarily practiced through the use of various insecticides [9,10,11]. Though controlling psyllid populations is essential to limiting initial infection and reinfection, it does not reduce the long-term effects of HLB [10,12]. Antibiotic applications (to directly control *C*Las) are also often incorporated by citrus growers [13,14]. By 2016, emergency exemptions led to the approval of streptomycin and oxytetracycline-based spray products [12]; the use of oxytetracycline as an injected antibiotic has now been approved with a 24(c) special local needs label [15]. However, antibiotic effectiveness is inconsistent [2,11], and their application is especially challenging for phloem-limited pathogens [16]. Further, while the use of insecticides and antibiotics have shown limited and highly variable effectiveness on HLB, their potential negative impacts on beneficial and non-targeted microorganisms and insects have been well documented [9,10,17]. Nevertheless, in the continuous attempt to address both immediate and long-term concerns of HLB, an array of diversified strategies (nutrition and water management, protective screening, breeding, small peptide molecules, gibberellic acid and antioxidants, biocontrol of vector, etc.) are being pursued in the attempt to manage orchards and maintain productivity [18,19,20]. Still, each strategy is dependent on a variety of factors, with its own set of limitations and varying degrees of effectiveness [21]. Thus, no available treatment options are sufficient for commercial HLB control [7,10]; they are, at best, only partially effective [22]. Irrefutably, there is an urgent need to develop sustainable and effective treatment options to prevent, reduce, or eliminate *C*Las from infected trees and alleviate the ongoing crisis in the citrus industry.

Plants produce thousands of active compounds known as secondary metabolites [23], which [24] provide an array of benefits, including stimulating immunity and protection from diseases [25]. Plant health is also influenced by the plant’s native microbiome [26,27]—its collective community of microorganisms. Endophytes are microorganisms (generally bacteria or fungi) that inhabit plant tissue without causing any apparent symptoms or disease [24,28,29], and they are most often classified as beneficial symbionts of their host plant. Like plants, endophytes also produce an array of bioactive secondary metabolites [29], which have demonstrated significant antimicrobial activity against a variety of organisms and which ultimately help to enhance host fitness [23,30]. Secondary metabolites produced by plants and endophytes have immense control potential against bacterial diseases [31]. Compared to their synthetic counterparts, these naturally produced compounds are less likely to be phytotoxic to host plants, and pathogens are unlikely to develop resistance [32,33]. Thus, crude plant extracts of medicinal plants, as well as the antagonistic activity of endophytic microorganisms (and their bioactive compounds) are being screened all over the world for their antimicrobial activity and biological control potential [31,34,35].

In addition to the benefits offered by naturally produced compounds, endophytes themselves offer additional unique characteristics that make them ideal candidates for biological control [35]; this is particularly true for long-living woody plant diseases [36], where endophytes have demonstrated significant biological control against numerous vascular wilt diseases [35]. Further, endophytes naturally reside in an ecological niche extremely similar to that of many phytopathogens [37] and thus offer a means of control that is potentially self-sustaining and able to spread on its own (after initial establishment). Control methods of this kind provide long-term disease suppression and reduce the need for chemical inputs [35]. Direct antagonism against *C*Las by another endophyte-dwelling microbe could lead to radical changes in the management of HLB.

Heavily HLB-infected groves containing apparently healthy citrus trees have been reported in Florida [38,39]. These trees are said to have slower rates of disease development and have been referred to as survivor [40] or escape [41] trees. Given that these trees have the same genotype and are grown under the same environmental conditions as their symptomatic neighboring trees, it has been suggested that the slow progression of disease development in survivor trees may be linked to their microbial communities [28,42]. Still, only a handful of studies have focused on the microbial communities of HLB survivor citrus trees [6,40,41,43,44].

Chemical treatments are notorious for their negative impacts on native microbial communities, including changes in microbial community structure, antimicrobial resistance, and killing of beneficial nontarget organisms [45,46]. In contrast, the unaltered microbial communities of organic agroecosystems are enriched with beneficial microorganisms [47]. Given that native microbiomes play key roles in plant health [27], the unaltered microbial communities of organic citrus orchards may be more likely to harbor native, beneficial, anti-*C*Las endophytes than conventional citrus orchards. Furthermore, within a plant’s microbiome, differences in microbial community assemblages exist naturally across different niches, e.g., the rhizosphere and endosphere [48], including certain endophytes being specific to certain plant structures [28]. Taking into consideration that its vector introduces *C*Las into the aerial portions of the tree, endophytes residing in the same aboveground ecological niche as *C*Las (and nearest to *C*Las inoculation points) may potentially harbor novel *C*Las antagonists, particularly in asymptomatic trees.

The purpose of this study was to assess potential anti-*C*Las crude extracts from selected medicinal plant species, as well as to isolate and identify potential *C*Las-antagonizing endophytes from a specifically targeted citrus microbiome (organically managed, asymptomatic, survivor citrus trees). In addition, we sought to assess the cell-free culture supernatants (CFCS) of the selected endophytic candidates for antimicrobial activity directly against *C*Las in vitro. The present study aimed to maximize the possibility of isolating novel and effective *C*Las-antagonists, carried out by targeting endophytes residing in unaltered microbial communities (organic orchards), from asymptomatic trees that are most likely to harbor beneficial microbes (survivor trees) and from stem tissue where direct *C*Las antagonism might exist naturally (aboveground endosphere). The discovery of a naturally derived antimicrobial compound or of an antagonistic endophyte effective directly against *C*Las could permit direct pathogen control (decreased titer), which would reduce or eliminate disease symptoms and increase plant productivity. Hence, a sustainable treatment option targeting *C*Las directly would elicit novel preventative and therapeutic management opportunities for citrus growers.

## 2. Materials and Methods

### 2.1. Sampling and Endophyte Isolation

Samples were collected from 2 USDA certified organic citrus orchards (Uncle Matt’s Organic Orchards) in Clermont, FL (Latitude: 28°38′54.4″ N Longitude 81°44′1.44″ W and Latitude: 28°36′38.9″ N Longitude: 81°45′00.3″ W); where neither orchard has been treated with synthetic pesticides nor antibiotics since at least 1998 and 1999, respectively. Stem samples were collected from nearly asymptomatic, survivor, sweet orange (Citrus × aurantium Sweet Orange Group) trees, choosing the healthiest looking branches. Presence of *C*Las was verified using conventional primers and quantitative polymerase chain reaction (qPCR).

Endophytes were isolated from a total of 45 branch segments using a standard isolation procedure [49,50], with minor modifications. Stem segments (approx. 60 mm long) were washed in mild liquid soap solution and rinsed thoroughly with running tap water. Surface sterilization was achieved by sequentially submerging each segment in 70% ethanol for 1 min and 0.625% sodium hypochlorite (10% Clorox, the Clorox company, Oakland, CA, USA) solution for 4 min, followed by 3 sequential immersions in sterile distilled water. Surface sterilization was verified by pressing disinfected stem segments along with aliquots of the sterile DI water used in the final rinse into fresh tryptic soy agar (TSA) and potato dextrose agar (PDA) medium plates, then incubating and observing for 10 days for any microbial growth. After surface sterilization, using a sterile scalpel, stem fragments were cut into pieces of approximately 5 mm in length. For isolation of fungal endophytes, 3 stem fragments were transferred onto 90 mm Petri plates containing dilute (1/10-strength) PDA amended with streptomycin (100 μg mL^−1^) and were incubated in the dark, for 5–10 days, at 25 °C. For isolation of bacterial endophytes, three 5 mm stem fragments were ground using a mortar and pestle with 1 mL of sterile DI water to obtain a homogenate solution; 100 µL of the homogenate solution was transferred onto 90 mm TSA Petri plates containing cycloheximide (100 μg mL^−1^) and were incubated for 5–10 days at 28 °C. Isolates were differentiated based on morphology (colony shape and color), pigmentation and growth rates, and each distinct bacterial and fungal colony was subcultured for isolation using the streak plate or hyphal tip method. Bacterial cultures exhibiting extensive colony spreading and/or biofilm growth were further subcultured on TSA amended with 75 μg mL^−1^ of p-nitrophenylglycerol (NPG) for inhibition of surface colony spreading [23]. Once pure cultures were confirmed for each endophyte, morphological characteristics of all isolates were recorded.

### 2.2. Bacterial Strains and Culture Conditions

*Sinorhizobium meliloti* (*S. meliloti*) strain 1011 was obtained from the USDA ARS National Rhizobium Germplasm Collection (Beltsville, MD, USA). The strain was grown and periodically subcultured on yeast extract mannitol agar (YMA) and incubated at 28 °C for 48 h. *Liberibacter crescens* (*L. crescens*) DSM 26877 was obtained from DSMZ (Leibniz Institute DSMZ-German Collection of Microorganisms and Cell Cultures, Braunschweig, Germany). The strain was grown in BM7 liquid media and BM7 agar, prepared as described by [51], incubated at 28 °C and subcultured every 7–10 days. Liquid cultures were maintained by incubation at 150 rpm in an incubator shaker. All potential endophytic antagonists used in inhibition assays were grown on agar medium (TSA or PDA), subcultured regularly, and kept under their established optimum conditions.

### 2.3. Dual Culture Assay

Initial screening of potential *C*Las antagonistic endophytes was assessed by means of the dual culture assay as described by [52] with some modifications. All dual cultures were streaked with *S. meliloti*, a phylogenetically related culturable surrogate for *C*Las [53,54]. Endophytic antagonists were assessed by streaking bacterial isolates approximately 30 mm from the edge of 90-mm TSA Petri plates, and fungal antagonists by transferring small segments (<3 mm) of mycelium to 90-mm PDA Petri plates, approximately 30 mm from the edge. *Sinorhizobium meliloti* was suspended to OD_600_ = 0.5 for consistent comparisons across all co-cultures, and a loopful of suspension was streaked approximately 30 mm away from the opposite edge of each Petri plate, resulting in each endophyte and *S. meliloti* growing parallel to each other approximately 30 mm apart. Triplicate dual-culture plates were prepared for each pure isolate, and all plates were incubated at 28 °C for approximately 12–24 h (depending on growth rate), followed by an additional 24-h incubation at room temperature. Antagonism of the endophytes was evaluated by comparing the growth of *S. meliloti* in the presence of each endophyte to that of the growth of *S. meliloti* when streaked alone (control plates). Inhibition was then categorized into 3 groups: no inhibition (same amount of growth as seen on control plates), moderate inhibition (about half the growth seen on control plates), and complete inhibition (no growth of *S. meliloti*). Isolates showing consistent inhibitory results (moderate or complete inhibition) on all 3 replicates were considered potential *C*Las antagonists and thus selected for further evaluation.

### 2.4. Agar-Well Diffusion Assay

The cell-free culture supernatants of endophytic isolates demonstrating antagonistic activity (moderate and/or complete inhibition) against *S. meliloti* on the dual culture assay were produced and used in the agar-well diffusion assay. Antagonistic endophytes were inoculated into 30 mL of broth media (20% Tryptic Soy Broth (TSB)) and incubated at 27 °C on a rotary shaker at 150 rpm for 48 h. Cells were removed by centrifugation at 4500 rpm for 35 minutes at 4 °C; the supernatant was transferred for membrane filtration (0.20 µm) and stored at −20 °C for later use. Antimicrobial activity of the CFCS was evaluated against a surrogate organism, *L. crescens*, the only cultured species in the *Liberibacter* genus [55], using the in vitro agar-well diffusion assay as described in [56], with modifications. Briefly, 100 µL of *L. crescens* culture (containing 1 × 10^8^ cells mL^–1^) was uniformly spread on BM-7 media with a sterile cell spreader. A 6 mm diameter well was punched aseptically with a sterile cork borer, and 100 µL of the CFCS sample or sterile DI water control was introduced into the wells. All plates were then incubated at 28 °C for 15 days, and the diameters of the resulting zones of inhibition (including the 6 mm diameter well) were measured in millimeters. Triplicate plates were prepared for each isolate, and the average zones of inhibition (diameter) were calculated and reported as follows: less than 7 mm was considered no inhibition (−), between 7 and 15 mm was considered moderate inhibition (+), and more than 15 mm was considered strong inhibition (++).

Commercial extract samples from 13 medicinal plant species were selected based on the literature indicating significant antimicrobial activity against Gram-negative bacteria (similar to *C*Las). The following 13 crude plant extracts were assessed: wormwood (*Artemisia annua*), nibima (*Cryptolepis sanguinolenta*), Christmas bush (*Alchornea cordifolia*), cobbler’s pegs (*Bidens pilosa*), teasel (*Dipsacus fullonum*), cat’s claw (*Uncaria tomentosa*), oregano (*Origanum vulgare*), thyme (*Thymus vulgaris*), cinnamon (*Cinnamomum aromaticum*), turmeric (*Curcuma longa*), basil (*Ocimum tenuiflorum*), bearded lichen (*Usnea longissima*), and banderol (*Otoba parvifolia*). Initial screening for the in vitro antimicrobial activity of the 13 plant extracts was evaluated by agar-well diffusion assay using BM7 media and the surrogate organism *L. crescens*. One hundred microliters of *L. crescens* culture (containing 1 × 10^8^ cells mL^–1^) was inoculated on BM7 media, and a 6 mm diameter well was punched aseptically through the center of each plate and allowed to diffuse for ~1 h. Extracts were serially diluted in sterile DI water, where 100%, 10%, and 1% (*v*/*v*) were assessed, and 100% sterile DI water was used as the control. One hundred µL of each treatment was introduced into the wells (in triplicates), and plates were incubated at 28 °C for 15 days; and inhibition zones were measured using the same scale as was used for the bacterial CFCS.

### 2.5. Leaf-Disk Assay

Plant extracts demonstrating strong antimicrobial activity (inhibition zone) against *L. crescens* were screened for in vitro activity against *C*Las using the leaf-disk assay. A total of 21 HLB-symptomatic leaves were collected from 3 rough lemon (*Citrus jambhiri*) trees (seven per tree) from the U.S. Horticultural Research Laboratory (USHRL), Fort Pierce, FL. Leaves were washed with deionized water and mild soap (Dawn Ultra, Procter & Gamble, Cincinnati, OH, USA). As a wide spectrum antibiotic that has been used experimentally to treat HLB [10], streptomycin (0.5 mM) was assigned as the positive control. Two hundred μL of each plant extract treatment (at 1%) and 0.5 mM streptomycin were added to their respective wells (7 replicates of each treatment per 96-well plate). To reduce variability associated with *C*Las, this assay used a cross-comparison of disks taken from the same leaf as a blocking factor [57]. Using a sterile biopsy punch, 4 mm leaf disks were punched along the midrib of each leaf, beginning from the base of the leaf and moving out. The first and last disks of each leaf were placed in 1.5 mL micro-centrifuge tubes and flash frozen with liquid nitrogen to be used for initial values. All other disks were placed in their respective treatment solution in a randomized order (resulting in 7 treatment replicates for each tree), and all disks were left to incubate at room temperature for 48 h.

After incubation, leaf disks were transferred to 1.5 mL micro-centrifuge tubes, where they were frozen in liquid nitrogen and ground to a fine powder. Two hundred μL of Rnase A was added to TE buffer (Fluka^®^ Tris-EDTA buffer solution pH 8.0 #93283-500ML, Sigma-Aldrich, St. Louis, MO, USA), and 200 μL of this solution was added to each micro-centrifuge tube. Samples were transferred to a 96-well tube rack and incubated for 15 min at 37 °C. A phenol-based extraction was performed [58], where pellets were resuspended in 52 μL of nuclease-free water, and nucleic acid concentrations were quantified using 2 μL on a Nanodrop spectrophotometer; the remaining 50 μL samples were diluted to 100 ng μL^−1^ for qPCR use.

Quantitative PCR (qPCR) was conducted with Taqman fast chemistry using GoTaq qPCR Master Mix. Each reaction well received 10 μL of master mix, 4 μL of nuclease free water, 2 μL of primer, and 4 μL of template sample (100 ng/μL). Primers for *C*Las 16S rDNA and citrus dehydrogenase were used for quantification by qPCR; sequences are reported in Table 1. The 96-well plates were loaded into the AB 7500 Real-Time PCR System (Thermo fisher, Waltham, MA, USA), and the program was run for Taqman signal with the following parameters: holding stage at 95 °C for 2 minutes, and cycling stage (40×) at 95 °C for 15 s, followed by 62 °C for 60 s. Statistical analysis was conducted with the SAS with JMP genomics package. One-way ANOVA tests were performed to compare statistical means. The Hodges–Lehmann–Sen estimator was used to show significance between treatments. Data were considered significant when *p* < 0.05.

### 2.6. Psyllid Homogenate Assay

Bacterial CFCS having strong antimicrobial activity against *L. crescens* (on diffusion assay) were screened for in vitro activity against *C*Las using the psyllid homogenate assay [59], a protocol designed specifically to test the effectiveness of potential therapeutic agents against *C*Las. *C*Las-infected (*C*Las+) ACP were provided by USHRL, Fort Pierce, FL, from colonies maintained on *C*Las+ citrus, with *C*Las levels monitored monthly by randomly sampling adult insects. The negative control was a sterile 20% TSB solution (the medium the endophytes were grown in), and a solution of 0.1% Triton-X 100 in 20% TSB was used as the positive control (previously found to have nearly complete *C*Las cell disruption, with a lysis effect between 85–98%) [59].

One hundred and fifty *C*Las+ ACP were euthanized with 95% EtOH. Psyllid bodies were transferred (in groups of 30) into centrifugal filter tubes (0.65 µm spin columns) and centrifuged at 1000× *g* for 5 s to remove EtOH. Flow-through was discarded and the spin column was washed with 600 μL of sterile deionized water, which was then centrifuged again (same as previous parameters). Filter units were transferred to new sterile centrifuge tubes, where 150 μL of isolation buffer (21 mM KCl, 36 mM NaCl, 15 mM MgSO_4_, a 1 mM phosphate buffer at pH 7 (K_2_HPO_4_  +  KH_2_PO_4_) and 1% glycerol, optimized for maximum recovery of intact *C*Las cells) was added. A plastic pestle and motorized handle were used to gently macerate the psyllids in each tube (to expose hemolymph), which were then centrifuged for 10 min at 16,000× *g*. Each filter was discarded, and the remaining pellets were resuspended (by pipetting) and pooled, resulting in a single 750 μL *C*Las containing homogenate solution. Two 5 μL samples were removed and stored at −20°C for initial *C*Las titer assessment, and the remaining homogenate was aliquoted into 90 μL samples, to which 10 μL of the assigned treatments (controls and bacterial CFCS) was added. Negative (dilute TSB) and positive (Triton-X 100) controls were the same as described above. Treatments and controls were further divided into individual 5 µL replicates in 8-tube strips of 0.2 mL micro-centrifuge tubes and incubated at room temperature for 4 h.

After incubation, each 5 µL treatment sample was supplemented with 1 µL of 0.15 Mm of PMAxx (a proprietary modified propidium monoazide (Biotium, Fremont, CA, USA)) for a final concentration of 25 µM, and the no-PMAxx controls received 1 µL of sterile nuclease-free water instead. All samples were mixed through tap-spin, incubated at room temperature for 5 min, and placed in an open bottom rack on the Glo-Plate blue LED illuminator (Biotium) and exposed at 465 nm for 15 min. Immediately afterward, DNA was extracted from all samples [58] using 45 μL of lysis buffer (0.1% SDS, 0.05% Tween-20 and Tris-EDTA pH 8.0 Sigma-Aldrich #93283), and 50 μL of phenol was added to each isolation. The remaining DNA pellets were then suspended in 50 µL of nuclease-free water and gently vortexed to dissolve the pellet.

Viability qPCR (v-qPCR, an assessment of intact, non-disrupted cells following initial incubation) was performed with the use of PMAxx and was conducted using Taqman fast chemistry. Each reaction well received 1 μL of DNA template, 0.5 μL of LLj primers, 8.5 μL of nuclease free water, and 10 μL of GoTaq qPCR master mix. *C*Las primers were the same as those previously mentioned (LLjs). The 96-well plates were briefly placed to tap-spin, loaded into the AB 7500 Real-Time PCR System, and set to the following: holding stage at 95 °C for 20 min and cycling stage at 95 °C for 3 min and 60 °C for 30 min. Antimicrobial control efficiency (represented by *C*Las disruption during incubation) was then assessed by measuring differences in Ct values within PMAxx and across (with control) treatments. Statistical analysis was conducted using SAS with the JMP genomics package. Data were compared using Wilcoxon non-parametric analysis and were considered significant when *p* < 0.05. Viability qPCR reactions resulting in an undetermined Ct were not included in the mean calculations.

### 2.7. Identification of CLas Antagonists

Pure cultures of endophytes demonstrating significant in vitro antimicrobial activity against *C*Las were sent to MIDI Labs, Inc. (Newark, DE, USA) for phylogenetic analyses by 16S rRNA gene sequencing. Identification was performed by Sanger sequencing of the first 500 base pairs of the 16S rRNA gene, and Sherlock™ DNA microbial identification system analysis software version 6.0 (MIDI, Inc., Newark, DE, USA) was used for sequence data analysis. The partial 16S rRNA gene sequences of each isolate were also used to construct a phylogenetic tree using the neighbor joining tree method.

## 3. Results

### 3.1. Dual Culture Assay

A total of 342 morphologically distinct endophytes (179 bacteria and 163 fungi) were isolated to pure cultures and tested for initial antimicrobial activity by means of the dual culture assay (Table 2). Each isolate was initially screened for antagonistic activity against the *C*Las-surrogate, *S. meliloti*. Control plates consisted of *S. meliloti* streaked alone (no competition), and results were categorized as no inhibition (−), moderate inhibition (+), or complete inhibition (++). Among the 342 screened isolates, 315 (92.1%) showed no antagonism, 20 (5.8%) showed moderate inhibition, and 7 (2.1%) showed complete inhibition against *S. meliloti*. All 27 endophytes that showed at least some activity (moderate or strong) were bacterial isolates. No inhibition was observed from any of the endophytic fungal isolates. Given that bioactive fungal endophytes have been reportedly isolated from citrus [14], it is probable that the specific methods used in our dual culture assay were not ideal for the initial screening of fungal endophytes. A modified or alternate method targeting fungal endophytes may reveal potential *C*Las-antagonistic fungal endophytes.

### 3.2. Agar-Well Diffusion Assay

The CFCS of the 27 bacterial isolates demonstrating at least some inhibition (moderate or complete) against *S. meliloti* were produced and assessed for antimicrobial activity against *L. crescens* using the agar-well diffusion assay (Table 2). Results demonstrate that 18 isolates (66.7%) showed no inhibition (−), 4 isolates (14.8%) showed moderate inhibition (+), and 5 isolates (18.5%) showed strong inhibition (++) against *L. crescens*. The five isolates demonstrating strong inhibition were B-9, B-17, B-24, B-25, and B-27, with the following mean zones of inhibition: 37 mm, 40 mm, 47 mm, 33 mm, and 35 mm, respectively. Only these five bacterial isolates demonstrating strong inhibition of *L. crescens* were considered potential HLB therapeutics and selected for in vitro evaluation directly against *C*Las.

Initial screening of the antimicrobial activity of the 13 selected plant extracts was evaluated at three different concentrations (100%, 10%, and 1%) against *L. crescens* using the agar-well diffusion assay (Table 3). Results showed that among the 13 screened extracts, five (Christmas bush (*Alchornea cordifolia*), oregano (*Origanum vulgare*), thyme (*Thymus vulgaris*), cinnamon (*Cinnamonum aromaticum*), and turmeric (*Curcuma longa*)) demonstrated at least moderate inhibitory activity (≥7 mm) against *L. crescens* at each of the three concentrations tested and thus were selected for further evaluation. The remaining eight plant extracts were eliminated and no longer considered potential therapeutics for HLB.

### 3.3. Leaf-Disk Assay

**Figure 1:**
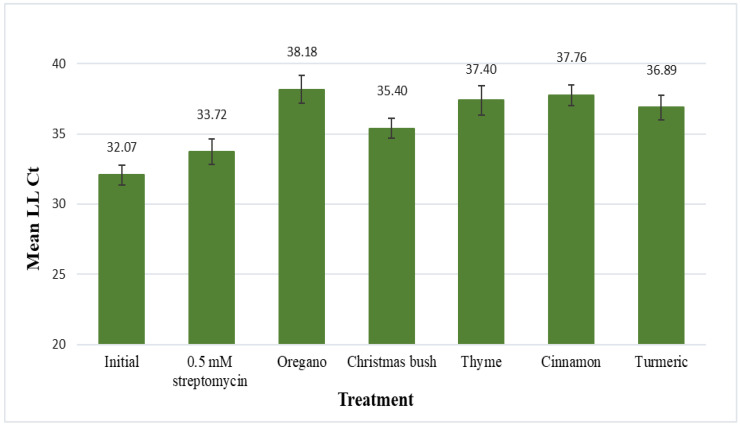
Leaf disks from HLB-symptomatic trees were submerged in 200 μL of treatment solution for 48 h. Treatments included a no-treatment control, streptomycin, oregano (*Origanum vulgare*), Christmas bush (*Alchornea cordifolia*), thyme (*Thymus vulgaris*), cinnamon (*Cinnamomum aromaticum*), and turmeric (*Curcuma longa*). Leaf disks were frozen in liquid nitrogen and ground to a fine powder for phenol extraction (Tris-EDTA buffer solution), followed by qPCR with *C*Las sequence-specific primers (LL, Las-Long). Results are shown as mean Ct values for *C*Las with standard error of the mean.

The five plant extracts demonstrating the best antimicrobial activity against *L. crescens* (oregano, Christmas bush, thyme, cinnamon, and turmeric) were evaluated for their antimicrobial effects on *C*Las using the leaf-disk assay (Figure 1). Results are displayed as mean LL Ct values (LLj primers) for *C*Las after 48 h of incubation in each treatment. Mean LL Ct values for the initial (no-treatment control) and for 0.5 mM streptomycin (positive control) samples were 32.07 and 33.72, respectively. No statistical difference was found between the initial and 0.5 mM streptomycin samples.

Mean LL Ct values for the five plant extracts were the following: 38.18 for oregano, 35.40 for Christmas bush, 37.40 for thyme, 37.76 for cinnamon, and 36.89 for turmeric; statistical differences were found between each of these and the no-treatment control, with *p*-values of 0.0007, 0.0039, 0.0035, 0.0002, and 0.001, respectively. Additionally, extracts of oregano, cinnamon, and turmeric were also found to be statistically different from streptomycin, with *p*-values of 0.0377, 0.0222, and 0.0279, respectively. Thus, all five plant extracts reduced *C*Las titer a statistically significant amount compared to untreated controls; three (oregano, cinnamon and turmeric) reduced *C*Las titer even more than the antibiotic streptomycin.

**Figure 2:**
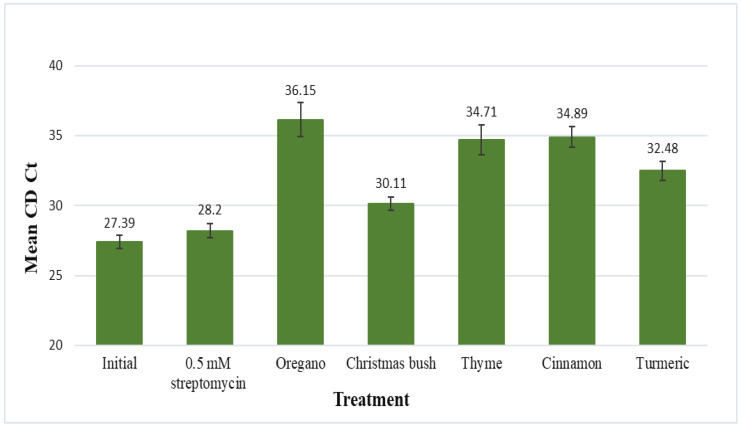
Leaf discs from HLB-symptomatic trees were submerged in 200 μL of treatment solution for 48 h. Treatments included a no-treatment control, streptomycin, oregano (*Origanum vulgare*), Christmas bush (*Alchornea cordifolia*), thyme (*Thymus vulgaris*), cinnamon (*Cinnamomum aromaticum*), and turmeric (*Curcuma longa*). Leaf discs were frozen in liquid nitrogen and ground to a fine powder for phenol extraction (Tris-EDTA buffer solution), followed by qPCR. Results are shown as mean Ct values for the citrus dehydrogenase endogene (CD Ct) with standard error of the mean.

Results for the citrus dehydrogenase endogene (CD Ct) are displayed as mean Ct values, which represent citrus cell quantities (inversely) after 48 h of incubation in each treatment (Figure 2). The mean CD Ct value for the initial (no-treatment control) was 27.39 and for 0.5 mM streptomycin was 28.20; no statistical difference was found between the two. Mean CD Ct values for each of the 5 plant extracts, oregano, Christmas bush, thyme, cinnamon, and turmeric, were 36.15, 30.11, 34.71, 34.89, and 32.48, respectively. Each of the 5 plant extracts were found to be statistically different than the initial and also statistically different than streptomycin. When compared to the no-treatment control *p*-values were the following: 0.0004 for oregano, <0.0001 for Christmas bush, 0.0001 for thyme, <0.0001 for cinnamon, and <0.0001 for turmeric; when compared to 0.5 mM streptomycin *p*-values were 0.0016 for oregano, 0.0014 for Christmas bush, 0.0009 for thyme, <0.0001 for cinnamon, and <0.0001 for turmeric. Additionally, discoloration of leaf disks was evident after incubation for each of the 5 plant extract treatments; thus, together with the statistical data suggests possible phytotoxicity to citrus from each of the 5 plant extracts.

### 3.4. Psyllid Homogenate Assay

The psyllid homogenate assay (in conjunction with v-qPCR) provided a means to measure the antimicrobial activity of the high-potential candidates directly against *C*Las (in vitro) in a cost-effective and time-efficient manner. The CFCS of the five bacterial isolates demonstrating the strongest bioactivity against the surrogate organisms were B-9, B-27, B-24, B-17, and B-25, and thus, these were assessed directly against *C*Las. Viability qPCR was performed with the use of PMAxx, a dye that effectively binds free DNA and allows differentiation between putatively live and dead cells [60]. For each treatment and control, nine replicates were supplemented with PMAxx, and three replicates were supplemented with nuclease-free water (no-PMAxx).

PMAxx, a photoreactive dye with a high affinity for DNA is not membrane permeable. Thus, when activated, the dye binds to DNA from membrane-compromised cells but is unable to bind with DNA from intact cells. Once bound, the modified DNA will no longer amplify in subsequent qPCR reactions, resulting in amplification only of cells that remain intact post treatment. Accordingly, PMAxx-treated samples represent putatively live (intact) *C*Las cells and no-PMAxx samples (amplification of all DNA) represent total *C*Las cells (intact and lysed). Thus, comparisons between no-PMAxx samples permits verification of initial *C*Las homogeny across all samples.

**Figure 3:**
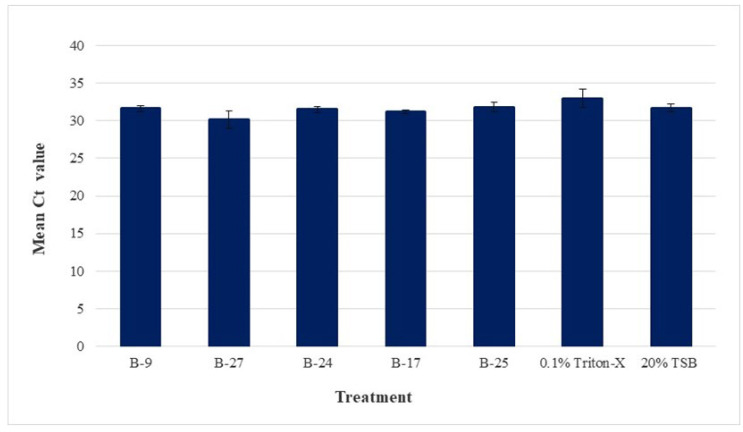
Mean Ct values for no-PMAxx control samples. *C*Las-infected psyllids were euthanized and macerated to prepare a *C*Las-containing homogenate. Treatments include the cell-free culture supernatants of five bacterial extracts (B-9, B-27, B-24, B-17, B-25), 0.1% Triton-X, and 20% TSB), where each was added to the homogenate aliquots and incubated for 4 h. Samples were supplemented with sterile nuclease-free water (in contrast to PMAxx treatments), exposed to blue LED light at 465 nm for 15 min, extracted for DNA, and taken for qPCR analysis of *C*Las 16S rDNA. Results are shown as mean Ct values with standard error of the means. No statistical difference was found between any of the no-PMAxx samples, demonstrating no variation in initial *C*Las DNA across all treatments and controls.

Figure 3 shows the average Ct value and standard error of the means for each treatment and control under the no-PMAxx condition. The average Ct values of the five bacterial CFCS (B-9, B-27, B-24, B-17, and B-25), 0.1% Triton-X, and 20% TSB were the following: 31.64, 30.15, 31.52, 31.16, 31.84, 32.97, and 31.70, respectively. No statistical difference was found between any of the no-PMAxx samples, thus confirming the initial homogeny of *C*Las across all samples as well as that the treatments themselves did not interfere with qPCR efficiency.

Given that PMAxx samples represent intact *C*Las cells and no-PMAxx samples represent total *C*Las cells, the change between PMAxx and no-PMAxx denotes the ratio of putatively living to dead *C*Las cells (ΔCt). Changes in the live/dead ratio or the PMAxx-treated sample (when all treatments begin with a homogeneous sample as in this trial) then show lysis activity of the treatment in rupturing cell membranes, allowing PMAxx to penetrate the cell. Further, a comparison of the live/dead ratio (ΔCt) of each treatment against the controls elucidates *C*Las lysing activity.

**Figure 4:**
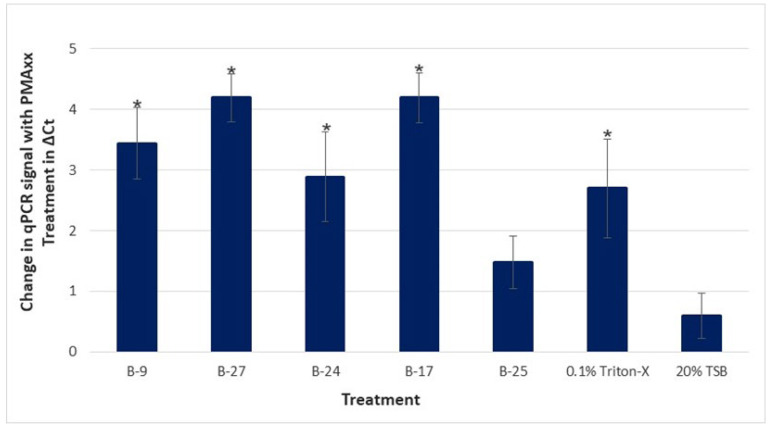
Difference in qPCR signal of living to dead *C*Las with PMAxx (proprietary modified propidium monoazide) treatment. Samples for treatment were prepared using a single homogenate of Asian citrus psyllid (ACP) from a *C*Las-positive colony. Treatments include the cell-free culture supernatants of five bacterial extracts (B-9, B-27, B-24, B-17, B-25), 0.1% Triton-X, and 20% Tryptic Soy Broth (TSB), where each was added to the homogenate aliquots and incubated for 4 h, with nine technical replicates per treatment. Samples were treated with PMAxx, followed by a DNA extraction and qPCR analysis of *C*Las 16S rDNA. The results are shown as the mean difference in Ct (ΔCt) of qPCR samples with and without PMAxx treatment with standard error of the means. A positive value represents an increase in Ct for the PMAxx-treated sample and a corresponding decrease in the viable cell count. * Samples are statistically different from TSB control by Wilcoxon non-parametric comparison (*p* < 0.05).

Figure 4 shows the difference between the mean Ct values of PMAxx and no PMAxx for each treatment and control (ΔCt). The mean ΔCt for Triton-X and TSB were 2.69 and 0.599, respectively; a significant difference (*p*-value of 0.024) was observed between the two. The mean ΔCt for B-9, B-27, B-24, B-17, and B-25 was 3.44, 4.19, 2.89, 4.20, and 1.48, respectively; statistical differences were found between four of these isolates and the TSB control: B-9 (*p*-value of 0.0118), B-27 (*p*-value of 0.0006), B-24 (*p*-value of 0.0141), and B-17 (*p*-value of 0.0006). This assay was replicated, and the results were consistent with that of trial 1. No statistical differences were found in the no-PMAxx samples, indicating no variation in initial homogenate. When comparing the live/dead ratio (ΔCt) of each treatment against the controls, a significant difference was again observed between 0.1% Triton-X and the TSB control. The only difference found in trial 2 was that statistically significant differences were found between the control and each of the five bacterial treatments, including B-25, which was not seen in trial 1.

Altogether, four of the five bacterial CFCS (B-9, B-27, B-24, and B-17) demonstrated statistically significant reductions in viable cells (greater ΔCt) on both trials. Although B-25 demonstrated the smallest ΔCt on both trials, given that statistical differences were found on at least one of the two trials, B-25 was still considered a potential anti-*C*Las agent, along with the additional four bacterial treatments. Further, it is also noteworthy to mention that no statistical differences were found (in either of the two trials) between any of the five bacterial CFCS and Triton-X, the positive control; as such, the effects of the bacterial treatments B-9, B-27, B-24, and B-17 were each statistically similar to the effect of Triton-X.

### 3.5. Identification of CLas Antagonists

**Figure 5:**
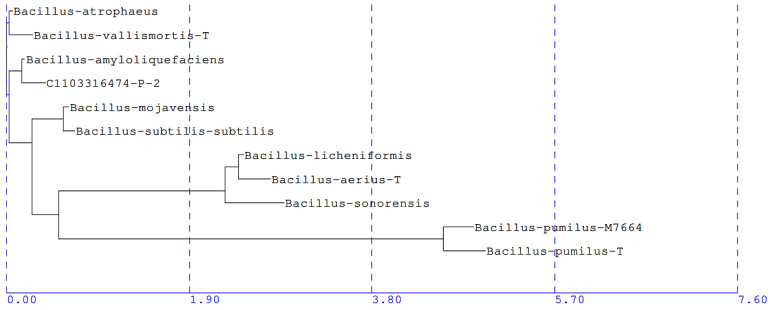
Phylogenetic tree of *C*Las antagonists, based on 16S rRNA sequences (accession number OR068131) of the five bacterial isolates demonstrating in vitro antagonism toward *C*Las. Sequences were aligned using Sherlock DNA database, and the phylogenetic tree was constructed using Neighbor Joining method. Each of the five effective bacterial isolates (C1103316474-P-2) were assigned a species-level match to *B. amyloliquefaciens* (accession number AB006920) with a percentage genetic difference of 0.28%.

The five bacterial isolates demonstrating significant reductions in intact *C*Las cells were sent for 16S rRNA gene sequencing, and alignment reports revealed that the 500 bp sequences of the five bacterial isolates (B-9, B-27, B-24, B-17, and B-25) were identical. This 16S rRNA gene sequence was deposited in GenBank under the accession number OR068131. Each isolate was assigned a species level match, reported as being most closely related to *Bacillus amyloliquefacie*ns (*B. amyloliquefaciens*) GenBank accession number AB006920, with a percentage genetic difference of 0.28%. The partial 16S rRNA gene sequence of these isolates was also used to construct a phylogenetic tree using the Neighbor Joining method (Figure 5).

## 4. Discussion

Strong initiatives toward sustainability and the increasing awareness of environmental concerns have significantly increased the demand for agricultural pest control agents described as biologicals (including plant-based products and antagonistic microorganisms). While research efforts have paved the way for the development and availability of many of these biologically derived products, advancements in the biological control sector of HLB are lagging, particularly with regard to direct antagonism. Seventeen years have passed since HLB was first detected in Florida; yet, despite tremendous efforts to combat the disease, HLB continues to devastate the citrus industry. Currently available management options consist primarily of insecticides for vector control and antibiotics for *C*Las control; while these chemicals are partially effective toward HLB, they are linked to several negative impacts on the environment and human health [61]. A naturally derived bioactive compound or an antagonistic endophyte demonstrating direct antimicrobial activity against *C*Las may well offer an indispensable, long-term, sustainable treatment option for the citrus industry, while also increasing consumer acceptance.

Given that *C*Las is a Gram-negative bacterium, plant extracts that had been previously documented for inhibition of other Gram-negative bacteria were reviewed; 13 crude extracts were initially screened against *C*Las surrogate *L. crescens*. The crude extracts of five medicinal plant species were considered high-potential anti-*C*Las agents and were thus evaluated directly against *C*Las (in planta) using a leaf-disk assay. In general, evaluating treatment efficacy from leaf-disk assays involving *C*Las is challenging due to the naturally occurring uneven distribution of *C*Las, resulting in variations of *C*Las titer along the plant. Here, lack of homogeneity impedes effective evaluations of treatments (especially those with low efficacy). Nonetheless, it has been reported that the use of multiple disks taken from the same leaf significantly reduces the random variability associated with *C*Las [57], and thus, this hindrance was addressed in our assay. Low initial infection rates are another hurdle in analyzing the efficacy of treatments, particularly due to the minimized effect potential. Our results were primarily affected by low quantities of initial *C*Las titer (LL Ct of 32.07) as well as a relatively short treatment window, which may also explain why no significant difference was found between streptomycin and the initial no-treatment control. Further, it has occasionally been reported that streptomycin has been non-effective against *C*Las [62]. Still, even under suboptimal conditions, each of the five plant extracts demonstrated statistically significant antimicrobial activity against *C*Las.

While these results are encouraging, results regarding the citrus endogene demonstrated that each of the five crude extracts (at concentrations of 1%) were also potentially phytotoxic to citrus. Nevertheless, considering the significant bioactivity seen against *C*Las in vitro, and considering that they were crude extracts, these results continue to present considerable potential. Various botanicals have already been reported as potentially beneficial agents for HLB management, with emphasis placed on their environmentally safe nature [20]. Thus, the five crude plant extracts of oregano, Christmas bush, thyme, cinnamon, and turmeric merit further exploration. Bioassay-guided fractionation analyses are needed to identify the biologically active compound/s against *C*Las, and subsequent in planta studies are required to assess the toxicity of those bioactive compound/s against citrus. That is, it may be that their bioactivity and phytotoxicity are triggered by different compounds. Still, while in their crude form, and at concentrations of 1%, oregano, Christmas bush, thyme, cinnamon, and turmeric should not be considered potential control agents for HLB. Similar phytotoxicity analyses on the citrus dehydrogenase endogene were performed on the five bacterial isolates showing strong anti-*C*Las activity (B-9, B-27, B-24, B-17, and B-25), and the results demonstrated no significant difference between the control and any of the bacterial CFCS, deeming the five bacterial CFCS as non-phytotoxic to citrus.

One of the major limitations to the development of effective treatments acting directly against *C*Las is that the pathogen remains unculturable. Although culturable surrogates have their own limitations, their close phylogenetic relationship to the unculturable pathogen provides unique opportunities for insight. As such, *S. meliloti* and *L. crescens* have been used as surrogates to screen a variety of antimicrobial compounds for potential activity against *C*Las [14,41,54,63]. In the present study, the process of selecting potential anti-*C*Las agents was accomplished by screening candidates sequentially, based upon phylogenetic distance from *C*Las. That is, initial screening of all candidates began (using the dual culture assay) with the more distant surrogate (*S. meliloti*), which belongs to the same family (Phyllobacteriaceae) as *C*Las and is relatively easy to manipulate in the laboratory. Candidates considered ineffective (no inhibition) against *S. meliloti* were eliminated, while candidates considered effective (showing inhibition) were advanced to assessments against *L. crescens*, the closest culturable surrogate of *C*Las [55]. Although *L. crescens* shares 94.7% 16S rRNA gene sequence with *C*Las [64], it is a slow-growing (8–10 days for first visible appearance) fastidious bacterium requiring a specific nutrient-rich growth medium (BM7) [51]; this presents various challenges to its use and maintenance in the laboratory. Correspondingly, *L. crescens* is unsuitable for co-culture assays, and thus, the agar-well diffusion assay was used for the second round of eliminations (using the CFCS of the antagonistic endophytes); only those demonstrating strong antimicrobial activity against *L. crescens* were deemed worthy of assessment directly against CLas in vitro.

Given that 92.1% of the initial isolates were eliminated during initial screening against *S. meliloti* and that 33.3% of those effective against *S. meliloti* were also effective against *L. crescens*, it is apparent that *S. meliloti* serves as an efficient surrogate for prescreening antagonistic candidates effective against *C*Las and should continue to be used in future studies. *S. meliloti* may also serve as an alternative to *L. crescens* altogether, especially for large-scale screening, where the substantial laborious work and costs associated with *L. crescens* may be prohibitive. Additionally, all five (100%) of the CFCS demonstrating strong inhibitory activity against *L. crescens* also demonstrated significant antimicrobial activity against *C*Las (at least on 1 trial). These results demonstrate the efficiency of *L. crescens* as a *C*Las-surrogate and, as such, are also encouraging for the potential anti-*C*Las agents that have been identified via *L. crescens* surrogacy [14]. Collectively, our approach of sequential eliminations verified the reliability and utility of surrogate organisms, particularly that of *S. meliloti* and *L. crescens.* Overall, a series of evaluations involving the sequential use of *S. meliloti*, followed by *L. crescens*, and finally *C*Las (in vitro), was found to be an effective technique in narrowing down and selecting high potential *C*Las antagonists.

Although the fastidious nature of *C*Las has impeded its growth in the laboratory, molecular methods involving assessments of cell membrane integrity have also been used to identify and quantify *C*Las presence [65]. Through the use of the psyllid homogenate assay and v-qPCR (with PMAxx), it was possible to assess and measure the antimicrobial activity of five bacterial CFCS directly against *C*La, in vitro. Here, we demonstrated that the CFCS from four bacterial isolates (B-9, B-27, B-24, and B-17) each caused significant reductions in intact *C*Las cells (by cell membrane disruption) in 4 h of incubation. Although the fifth bacterial CFCS (B-25) showed significant reductions in only one of the two trials, it was still considered a potential *C*Las biocontrol agent. Overall, our results also demonstrate that the psyllid homogenate assay may be readily employed for assessment of direct anti-*C*Las activity (specifically for cell membrane disruption) of potential therapeutic compounds. Furthermore, in addition to the aforementioned significance of the results, antimicrobials targeting cell membrane disruption present additional benefits in combating bacterial infections (compared to agents targeting intracellular systems). That is, cell membrane disrupting agents are likely to exert rapid bactericidal activity, with many of them using complex, multitargeted modes of action; thus, the ability of bacteria to develop resistance to membrane disrupting agents is limited [66,67].

The 16S rRNA sequencing and phylogenetic analysis revealed that each of the five effective bacterial endophytes has a gene sequence similarity closest to *B. amyloliquefaciens* (0.28% genetic difference). Although an exact threshold for species differentiation in bacteria is difficult to quantify, a gene sequence similarity threshold of 98.65% has been proposed [68]; thus, given our sequence similarity of 99.72%, it is likely that the five bacterial isolates are in fact *B. amyloliquefaciens* species. Furthermore, even though the 16S rRNA gene sequences of the five bacterial isolates were identical, each isolate growth shows morphologically distinct characteristics (colony shape and color), and thus, the five isolates are each likely different strains of the *B. amyloliquefaciens* species.

*Bacillus* species are ubiquitous, Gram-positive, spore-forming bacterium, which are widely recognized for their remarkable biological function against numerous plant pathogens, accounting for about half of the registered biological control agents [69]. Their use in agriculture includes plant growth promotion [70,71] and a broad spectrum of antimicrobial activity [61,72], as well as a notable capacity for biological control against vascular plant diseases [61]. Correspondingly, *B. amyloliquefaciens* has demonstrated significant antibiotic and antifungal activity against numerous plant pathogens [61,69,73] and has in fact been described as one of the most promising bacteria for plant growth promotion [71]. Additionally, *B. amyloliquefaciens* has demonstrated a distinguished ability to colonize both the rhizosphere and the phyllosphere, where it has outgrown plant pathogens through the induction of systemic defenses, competition, or direct antagonism [74].

With regards to citrus, the pervasiveness of *Bacillus* is no different. *Bacillus* species are native [27] and dominant [14] residents of the citrus microbiome. As for HLB, healthy and asymptomatic trees (as compared to symptomatic) have been found to harbor higher frequencies of certain beneficial *Bacillus* species (such as *B. subtilis*); thus, it has been suggested that certain *Bacillus* species play a role in enhancing HLB resistance [27] and improving the immune responses (increased photosynthesis and enhanced expression of resistance-related genes) of citrus trees [70,72]. In addition to the indirect benefits of *Bacillus* on HLB, potential anti-*C*Las *Bacillus* candidates have also been reported, due to antimicrobial activity against *C*Las-surrogates [14,41]. However, to the best of our knowledge, the direct effect against *C*Las from a *Bacillus*-based treatment has not been reported.

The primary challenge associated with an HLB treatment based on the direct antagonism of *C*Las is that an effective treatment requires direct entry into the phloem [14]; a successful biocontrol agent may need to not only enter the phloem but colonize it as well [75]. Although direct phloem entry has not been reported (to the best of our knowledge), we propose that Bacillus-derived biomolecules may permeate from the xylem and associated cells into the phloem. Further, endophytes are considered ideal candidates for biological control precisely for this reason: their marked ability to colonize an ecological niche similar to that of plant pathogens (including vascular pathogens) [35]. Accordingly, in order to increase the likelihood of recolonization, the present study aimed to isolate endophytes residing in a niche similar to that of *C*Las (citrus stem tissue), though it may not have been specifically from the phloem alone. Moreover, the isolated endophytes were native to citrus (in an organic orchard), and thus, the reintroduction of these endophytes into citrus groves would be unlikely to have any environmental risks [37]. In addition to these advantages, each of the isolates demonstrating significant activity against *C*Las are likely *B. amyloliquefaciens* species; given that *Bacillus* have the ability to colonize internal plant tissue [37] and that *B. amyloliquefaciens* are among the most successful biological control agents [74], our results are encouraging.

Thus, the five strains of *B. amyloliquefaciens* identified in the present study hold significant potential to become the first sustainable anti-*C*Las treatment agents for HLB control. In addition, given that *B. amyloliquefaciens* may induce the immune responses of HLB-infected citrus [70,72] and that in the present study five strains of *B. amyloliquefaciens* demonstrated strong antimicrobial activity directly against *C*Las, it is possible that *B. amyloliquefaciens* may actually provide dual-benefits (both direct and indirect) simultaneously to their citrus host trees. Nevertheless, given the complexities of the *Bacillus* phylogeny and the limitations of species identification based on 16S rRNA [76], as well as the fact that numerous strains of antimicrobial-producing *B. amyloliquefaciens* species have already been documented, additional sequence analyses (whole genome) are needed for complete taxonomic identification of each of these five anti-*C*Las isolates.

Huanglongbing has led to permanent changes in the management practices of citrus globally. Since Florida citrus trees are already infected, efficient management requires effective and sustainable treatment options to help infected trees maintain health and productivity. To accomplish this, citrus growers should have access to a diverse set of effective HLB prevention and mitigation tools. As such, endophytes offer remarkable potential for the discovery and development of novel anti-*C*Las therapeutics with multiple modes of action to reduce resistance. Further, an endophyte-based bioactive product for biological control of HLB would constitute a novel sustainable treatment option for citrus growers, promoting long-term disease suppression and reducing the need for chemical inputs.

## 5. Conclusions

The availability of sustainable HLB management options is critical to the survival of the citrus industry. Still, despite extensive efforts, citrus production continues to plummet. The development of effective treatment options has been hindered by the fastidious nature of the *C*Las pathogen, limiting experimental methods primarily to the use of surrogate organisms and in planta studies. In the present study, the sequential use of two surrogate organisms led to the selection of five high-potential anti-*C*Las candidates, which were evaluated directly against *C*Las in vitro, under a v-qPCR-based psyllid homogenate assay. Here, we measured the effectiveness of the antimicrobial activity (produced by the endophytic isolates) against *C*Las. The CFCS of five endophytic strains of *B. amyloliquefaciens* were each found to statistically reduce the number of intact *C*Las cells when compared to the control. While these results are promising, they entail the need for further exploration. Future studies require the identification and characterization of the bioactive fractions in the CFCS as well as in planta analysis of both the bacterial suspensions and their bioactive fractions. All in all, stems from organically managed survivor citrus trees were found to harbor *C*Las-antagonistic endophytes as part of their aboveground microbiome. As such, these naturally derived anti-*C*Las agents hold significant potential for HLB management, potentially increasing yield and productivity while reducing environmental impacts. Finally, an effective anti-*C*Las agent capable of entering the host’s vascular tissue could provide a unique opportunity for growers to become more sustainable in their management practices and ameliorate the crisis of the citrus industry. 

## Figures and Tables

**Table 1:** Primers and probe sequences for *C*Las 16S rDNA (LLjs) and citrus dehydrogenase (CD).

ID	Target	Type	Sequence
LLjs-F	*C*Las 16S rDNA	Forward	5′-GCATGGAAACGTGTGCTAATAC-3′
LLjs-R	*C*Las 16S rDNA	Reverse	5′-AGGCTCTTACCCTACCAACTA-3′
LLjs-Probe	*C*Las 16S rDNA	Probe	5′/65-FAM/TTGGAGAGA/ZEN/GATGAGCCTGCGTTG/3IABkFQ/-3′
CDjs-F	citrus dehydrogenase	Forward	5′-GCAGCAGTTTCTTTGTCCTTATC-3′
CDjs-R	citrus dehydrogenase	Reverse	5′-CCAAGGAGAAGAAGGGCATATT-3′
CDjs-Probe	citrus dehydrogenase	Probe	5′-/5Cy5/ACCACCCAAAGTCTGAGGACGAAA/3IAbRQSp/-3′

**Table 2:** Assessment of the antagonistic activity of citrus endophytes against *Sinorhizobium meliloti* (*S. meliloti*) and *Liberibacter crescens* (*L. crescens*) in vitro. Each isolated culture, B-1 to B-179 (bacterial) and F-1 to F-163 (fungal), was subject to sequential screening of the dual culture assay followed by agar-well diffusion assay. The dual culture assay was performed by streaking each endophyte approximately 30 mm from a parallel *S. meliloti* streak on tryptic soy agar medium. Isolates showing antagonistic activity were then assessed against *L. crescens* using 100 µL of their cell-free culture supernatants (CFCS) on the agar-well diffusion assay. Inhibition of colony growth of *S. meliloti* in dual culture was categorized as no inhibition (−) when having growth similar to that of control plates, moderate inhibition (+) when having ~50% less growth than control plates, or complete inhibition (++) when having no growth of *S. meliloti*. The growth inhibitory activity of CFCS against *L. crescens* was measured as average diameter in zones of inhibition from three replicate experiments and categorized as <7 mm or no inhibition (−), between 7 and 15 mm or moderate inhibition (+), and >15 mm or strong inhibition (++).

Isolate Number	Inhibition of *S. meliloti* in Dual Culture Assay	Inhibition of *L. crescens* by CFCS in Diffusion Assay
B-1	+	−
B-2	+	−
B-3	+	−
B-4	+	−
B-5	+	−
B-6	++	+
B-7	+	−
B-8	+	−
B-9	++	++
B-10	+	−
B-11	+	−
B-12	+	−
B-13	+	+
B-14	+	−
B-15	+	−
B-16	+	−
B-17	++	++
B-18	+	−
B-19	+	−
B-20	+	+
B-21	+	−
B-22	++	+
B-23	+	−
B-24	++	++
B-25	++	++
B-26	+	−
B-27	++	++
B-28 to B-179	−	N/A
F-1 to F-163	−	N/A

**Table 3:** Assessment of the antimicrobial activity of 13 medicinal plant extracts against *Liberibacter crescens* (*L. crescens*) in vitro. Each plant extract was tested by agar-well diffusion assay at three concentrations (100%, 10%, and 1% (*v*/*v*) in sterile DI water). One hundred microliters of *L. crescens* culture (1 × 10^8^ cells mL^–1^) was inoculated to create a uniform lawn on BM7 media, where 6 mm diameter wells in the plate centers were filled with 100 µL of each treatment. Growth inhibitory activity of each treatment was measured as average zones of inhibition from three replicate experiments and categorized as <7 mm or no inhibition (−), between 7 and 15 mm or moderate inhibition (+), and >15 mm or strong inhibition (++).

Plant Species		Concentration		
Scientific Name	Common Name	100%	10%	1%
*Artemisia annua*	Wormwood	++	+	−
*Cryptolepis sanguinolenta*	Nibima	+	−	−
*Alchornea cordifolia*	Christmas bush	++	+	+
*Bidens pilosa*	Cobbler’s pegs	−	−	−
*Dispsacus fullonum*	Teasel	−	−	−
*Uncaria tomentosa*	Cat’s claw	−	−	−
*Origanum vulgare*	Oregano	++	++	+
*Thymus vulgaris*	Thyme	++	+	+
*Cinnamomum aromaticum*	Cinnamon	++	++	+
*Curcuma longa*	Turmeric	++	++	+
*Ocimum tenuiflorum*	Basil	+	−	−
*Usnea longissima*	Bearded lichen	+	+	−
*Otoba parvifolia*	Banderol	−	−	−

## Data Availability

The original contributions presented in the study are included in the article, and further inquiries can be directed to the corresponding author.
